# Hypoparathyroidism and Fahr’s syndrome: case series

**DOI:** 10.1590/2175-8239-JBN-2020-0243

**Published:** 2021-05-17

**Authors:** Anna Catarina Gatzk de Arruda, Amanda Carolina Damasceno Zanuto Guerra, Carlos Henrique Pessoa, Guilherme Figueiredo Marquezine, Vinicius Daher Alvares Delfino

**Affiliations:** 1 Universidade Estadual de Londrina, Departamento de Endocrinologia e Metabologia, Londrina, PR, Brasil.; 2 Universidade Estadual de Londrina, Departamento de Nefrologia, Londrina, PR, Brasil.; 3 Universidade Estadual de Londrina, Departamento de Clínica Médica, Londrina, PR, Brasil.

**Keywords:** Hypoparathyroidism, Basal Ganglia, calcification, Fahr’s syndrome, Hipoparatireoidismo, Gânglios da Base, calcificação, síndrome de Fahr

## Abstract

Hypoparathyroidism (HP) is a rare metabolic disorder and causes hypocalcemia because parathyroid hormone secretion is inadequate to mobilize calcium from bone and reabsorb calcium from kidney and gut. Anterior neck surgery is the most common cause of acquired HP and autoimmune HP is the next most common form in adults. The duration, severity, and rate of development of hypocalcemia determine the clinical presentation. A variety of organs can be affected by calcification, more frequently kidneys, but also joints, eyes, skin, vasculature, and other organ systems and, although rarely seen, intracerebral calcifications. We report four cases of bilateral basal ganglia calcifications (BGC) also known as Fahr’s syndrome related to hypoparathyroidism. Fahr’s syndrome is characterized by bilateral symmetrical calcification of areas of the brain that control movements including basal ganglia, thalamus, and others; it is a rare inherited or sporadic neurological disorder with a prevalence of less than 1/1.000.000. Main symptoms related to bilateral BGC include extra-pyramidal and cerebellar disorders, cognitive impairment, epileptic seizures, and psychiatric changes. BGC has been established as a possible outcome of HP. Its prevalence, demonstrated in the HP cohorts, varied significantly from 12 up to 74%. Currently, computed tomography (CT) is the most valuable method for diagnosis. The treatment include symptomatic support and identification of causes, but there is no specific treatment limiting the progression of calcification in the basal ganglia. Especially in HP, an early treatment can prevent calcification and neurophysiological disorders.

## Resumo

O hipoparatireoidismo (HP) é um distúrbio metabólico raro e causa hipocalcemia, pois a secreção do paratormônio é inadequada para mobilizar o cálcio dos ossos e reabsorver o cálcio dos rins e intestino. A cirurgia cervical anterior é a causa mais comum de HP adquirido. O HP autoimune é a segunda causa mais comum em adultos. A duração, gravidade e taxa de desenvolvimento da hipocalcemia determinam a apresentação clínica. Diversos órgãos podem ser afetados pela calcificação, mais frequentemente os rins, mas também articulações, olhos, pele, vasculatura e outros órgãos e, embora raramente vista, calcificações intracerebrais. Relatamos quatro casos de calcificações dos gânglios da base (CGB) bilaterais, também conhecidas como síndrome de Fahr relacionadas ao hipoparatireoidismo. A síndrome de Fahr é caracterizada pela calcificação bilateral simétrica de áreas do cérebro que controlam os movimentos incluindo os gânglios da base, tálamo e outros; é um distúrbio neurológico raro, hereditário ou esporádico, com uma prevalência 1/1.000.000. Os principais sintomas relacionados à CGB bilateral incluem distúrbios extrapiramidais e cerebelares, comprometimento cognitivo, convulsões e alterações psiquiátricas. A CGB foi estabelecida como uma possível consequência do HP. Sua prevalência, demonstrada nas coortes de HP, variou significativamente de 12 a 74%. Atualmente, a tomografia computadorizada (TC) é o método mais valioso para o diagnóstico. O tratamento inclui suporte sintomático e identificação de causas, mas não há tratamento específico limitando a progressão da calcificação nos gânglios da base. Especialmente no HP, um tratamento precoce pode prevenir calcificações e distúrbios neurofisiológicos.

## Introduction

Hypoparathyroidism (HP) is a rare metabolic disorder, with an estimated prevalence of 37 cases per 100.000 inhabitants in the United States^1^, characterized by hypocalcemia due to absence or deficient production of parathyroid hormone (PTH) by parathyroid glands^1^^-^ 3 or by resistance to PTH in its target tissues, a condition called pseudo-hypoparathyroidism (PHP)^1^. The hypocalcemia occurs due to an inadequate PTH secretion to mobilize calcium from bone, reabsorb calcium from the distal nephron, and stimulate renal 1α-hydroxylase activity; as a result, insufficient 1,25-dihydroxyvitamin D is generated for efficient intestinal absorption of calcium^4^.

Metabolic dysfunction in HP results in ectopic soft tissue calcifications^2^. A variety of organs can be affected by calcification, more frequently kidneys (as nephrolithiasis or nephrocalcinosis), but also joints, eyes, skin, vasculature and, although rarely seen, intracerebral calcifications^2^. We report four cases of bilateral basal ganglia calcifications (BGC), also known as Fahr’s syndrome.

### Case Series

#### case 1

**Figure 1:**
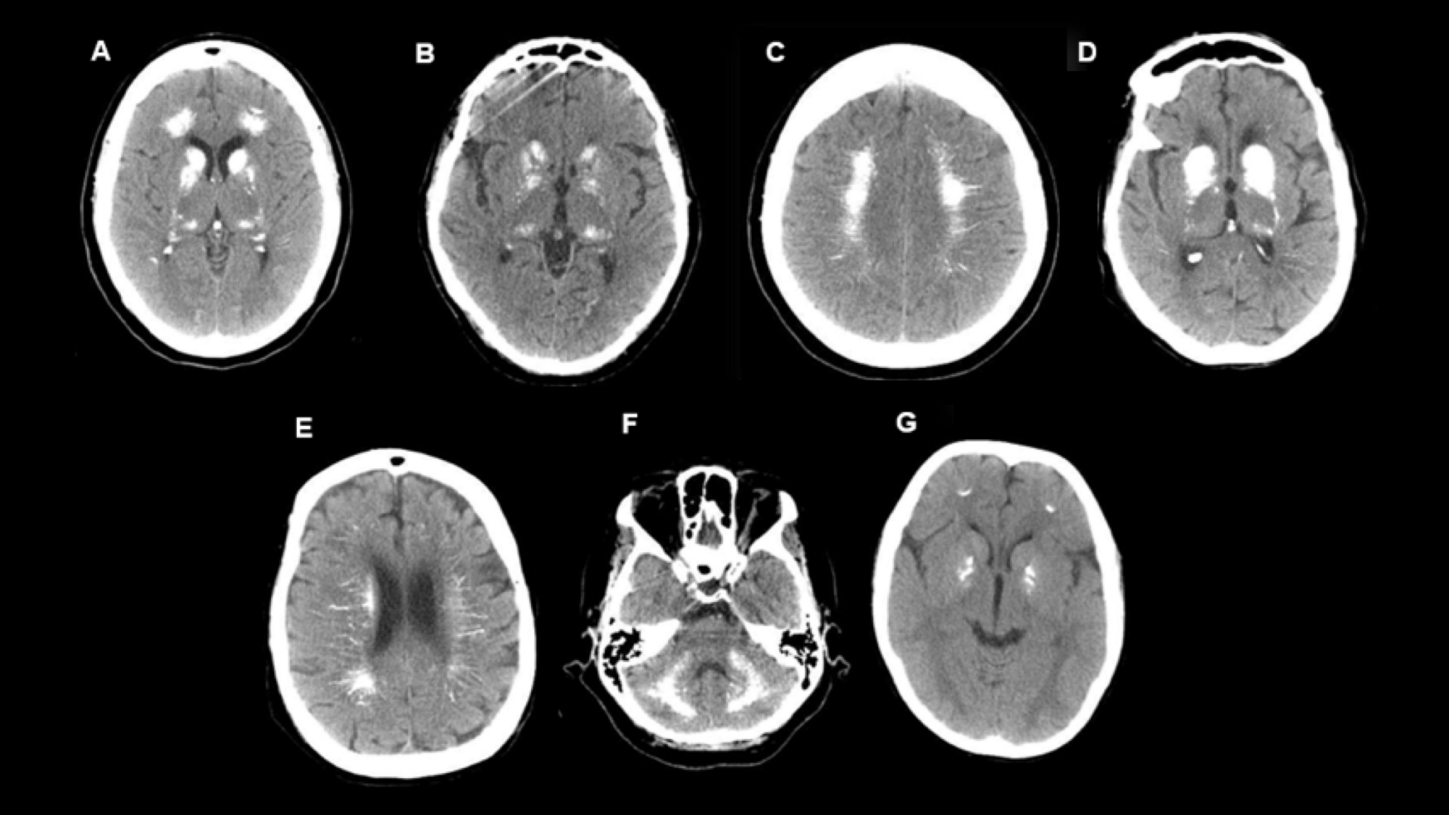
Cranial CT - Case 1 (A, B, and C): sparse white matter calcifications, base nucleus, thalamus, dentate nucleus, and cortical/subcortical regions in the occipital lobes. Case 2 (D and E): multiple symmetrical areas of calcifications in the supra/infratentorial deep gray matter and white matter in the perivascular and central pons territories. Case 3 (F): diffuse calcifications in the supratentorial white matter, base nucleus, thalamus, dentate nucleus, and some cortical/subcortical regions in the occipital lobes. Case 4 (G): calcifications in lentiform nucleus, heads of caudate nucleus, subcortical nucleus in frontal lobes, and cerebellar hemispheres.

A 61-year-old female with a history of multiple nodular goiter submitted to total thyroidectomy 32 years ago, developed hypothyroidism and HP after the procedure. Since then, she presented with chronic hypocalcemia besides adequate pharmacological therapy and medical orientations due to poor adherence to treatment. She had history of recurrent depressive disorder. She was hospitalized with acute signs of hypocalcemia as weakness, paresthesia, and presence of Chvostek sign. Laboratory evaluation showed low serum albumin‐corrected total calcium level, hyperphosphatemia, and low intact PTH (iPTH) level (Table 1). Cranial computed tomography (CT) revealed extensive bilateral symmetric supra and infratentorial calcifications (Figure 1). Hypocalcemia was corrected after administration of intravenous calcium and active vitamin D; she was discharged with oral medications.

**Table 1:** Clinical, radiologic and biochemical features

Data / Cases	Case 1	Case 2	Case 3	Case 4
**Age / Gender**	**61-year-old female**	**79-year-old female**	**75-year-old male**	**12-year-old boy**
HP etiology	Total thyroidectomy	Total thyroidectomy	Idiopathic	Type 1 APS
Cranial CT	Extensive bilateral symmetric **supra and infratentorial** calcifications	Extensive areas of symmetric basal calcifications in **deep gray matter supra and infratentorial white matter and pons**	Bilateral symmetric calcifications **supra and infratentorial, basal ganglia, thalamus, dentate nucleus and occipital lobes**	Bilateral symmetric calcifications on **lentiform and caudate nucleus, subcortical regions of frontal love and cerebellar hemispheres**
Total Calcium (8.5–10.0 mg/dL) / Ionized Calcium (1.17-1.32 mmol/L)	6.4 / -	- / 0.53	6.34 / -	6.2 / 0.31
Phosphorus (2.5-4.9 mg/dL)	6.6	High	5.5	5.8
iPTH (15-68.3 pg/nL)	Low	Low	Low (12.1)	Low (7.2)

#### case 2

A 79-year-old woman was admitted in the hospital with respiratory failure and mechanical ventilation assistance due to severe pneumonia. She underwent total thyroidectomy at 30 with no other known pathological medical history. She was used to take medications (named as “vitamins” by their relatives), that if missed, would cause symptoms of neuromuscular excitability as tetany. On admission lab tests, severe hypocalcemia, undetectable iPTH, and hyperphosphatemia were found besides leukocytosis and severe anemia (Table 1). She was diagnosed with post-surgical HP. Days after admission, family members brought her drug prescription that included levothyroxine, cholecalciferol, and calcium carbonate. During investigation, cranial CT showed extensive areas of symmetric basal calcifications in deep gray matter supra and infratentorial, white matter, and pons (Figure 1). Thorax CT with carcinomatous lymphangitis and a nodule in thyroid topography suggested neoplasia. There was no time to perform a cervical nodule biopsy due to the evolution to refractory septic shock and death.

#### case 3

A 75-year-old male was admitted in the hospital with a history of tonic-clonic seizures. That was the fifth episode of seizure in the previous year, with no others reported. His medical history included essential hypertension, dyslipidemia, and renal failure; there was no history of head and neck surgery or local radiation. On physical exam, vital signs and neurological exam were normal, and Chvostek and Trousseau signs were negative. Lab results showed hypocalcemia, hyperphosphatemia, low iPTH levels, and normal urinary calcium excretion (Table 1). Electrocardiogram presented prolonged QT interval. No abnormalities were found in thyroid and abdominal ultrasound. Cranial CT showed bilateral symmetric calcifications supra and infratentorial including basal ganglia, thalamus, and dentate nucleus and on occipital lobes (Figure 1). Diagnosis of HP was made, and treatment was established with intravenous calcium and calcitriol. He was discharged with oral medications.

#### case 4

A 12-year-old boy was admitted in the emergency unit, with a history of several tonic-clonic seizures and generalized edema initiated in the previous week. He had only one previous episode of febrile seizure when he was 3-month-old and reported upper airway infection two weeks before. His medical history included delayed neuropsychomotor development. Physical exam showed oral moniliasis. Laboratory tests showed low ionized calcium levels, low albumin corrected-calcium, hyperphosphatemia, and low iPTH levels; he also had nephrotic proteinuria (14.37 g/24 hours), low albumin level (1.0 g/dL, NR 3.4 to 5.0 g/dL), high thyroid-stimulating hormone (TSH 9.62 uUI/mL, NR 0.35 to 4.5 uUI/mL), and low free T4 level (0.66 ng/dl, NR 0.7 to 1.48 ng/dl). Urinary tract ultrasound identified a left atrophic kidney most related to previous pyelonephritis, without kidney stones or nephrocalcinosis. He also had bilateral cryptorquidism. Neck ultrasound was normal. On cranial CT it was identified bilateral symmetric calcifications on lentiform and caudate nucleus, subcortical regions of frontal lobe, and cerebellar hemispheres (Figure 1). Presumed diagnoses of type 1 APS (HP, hypothyroidism, and oral moniliasis) and nephrotic syndrome post infectious were made. Treatment with albumin and furosemide for kidney issues were realized, and intravenous calcium gluconate and calcitriol improved seizures episodes.

## Discussion

The lower PTH production may be induced by inadvertent removal or irreversible damage to the glands during surgical procedures of the anterior cervical region (as total thyroidectomy, parathyroidectomy, or radical neck dissection), being called post-surgical HP^1^^,^ 4, or by autoimmune, infiltrative, genetic, or irradiative causes^1^. Magnesium is essential for PTH secretion and activation of the PTH receptor, and its depletion or excess may cause hypocalcemia by inducing functional HP^4^. According to Clarke et al, anterior neck surgery is the most common cause of acquired HP, and responsible for about 75% of cases; less than 1-5% experience permanent HP, even though as many as 50% may develop transient HP^5^. Cases 1 and 2 represent post-surgical conditions that have become permanent HP, both related to thyroidectomy with inadvertent parathyroidectomy. The autoimmune is the second most common form of HP in adults^6^, and can occur in children and adolescents, isolated or as part of autoimmune polyendocrine syndrome (APS)^4^, as in case 4.

The diagnosis of HP occurs when the iPTH level is normal or inappropriately low in a patient with subnormal total or ionized calcium values, high serum phosphorus or at the high end of the normal range (NR), and after hypomagnesemia has been ruled out^4^^,^ 5. The duration, severity, and rate of development of hypocalcemia determine the clinical presentation^4^. HP most often presents with paresthesia, hyperreflexia, cramps, or tetany, but the disorder may manifest acutely with seizures, bronchospasm, laryngospasm, or cardiac rhythm disturbances^2^^,^ 6. Hyperreflexia is manifested by carpal spasms (Trousseau sign) and facial spasms (Chvostek sign). Other clinical manifestations include neuropsychiatric symptoms such as fatigue, hyperirritability, anxiety, and depression^2^^,^ 7. Of the four cases presented, three had been hospitalized with symptoms related to hypocalcemia, including tetany and seizures.

Fahr’s syndrome is characterized by bilateral symmetrical calcification of areas of the brain that control movements including basal ganglia, thalamus, dentate nucleus, cerebral cortex, cerebellum, subcortical white matter, and hippocampus^8^^,^ 9. It is a rare inherited or sporadic neurological disorder with a prevalence of less than 1/1.000.000, first described by German physician Karl Theodor Fahr in 1930^8^^,^ 10. This report presents four cases of Fahr’s syndrome with a wide variation of age and clinical presentation. Main symptoms related to bilateral BGC include extra-pyramidal and cerebellar disorders, cognitive impairment, epileptic seizures, and psychiatric changes^2^^,^ 9 ^,^ 11. It should be emphasized that only a small percentage of people with intracranial calcifications are symptomatic and, in most cases, it is an accidental discovery without any clinical implications^10^. BGC prevalence demonstrated in the HP cohorts varied significantly from 12 up to 74%. However, it should be noted that the estimated prevalence of BGC in the general population may achieve 12.5%^1^. BGC has been established as a possible outcome of HP. Pistacchi et al., in another case series, report that neuropsychiatric symptoms can be either the first or the most prominent manifestation ranging from mild concentration or memory impairment, personality and behavior changes, to psychosis and dementia^12^. In our report, all four cases were related to HP, two of them presented seizures and one psychiatric disorder that could be related to brain calcification or to hypocalcemia. Apart from HP and PHP, other metabolic, infectious and genetic diseases, as well as toxic conditions should also be considered as differential diagnosis in BGC^2^.

Mechanism of brain calcification in HP has been linked to long duration of hyperphosphatemia^7^^,^ 13 and high calcium-phosphate product, resulting from the disease itself and from long-term treatment with activated vitamin D and calcium^7^. High serum phosphate may also activate the inorganic phosphate transporter pit1 and result in the expression of osteogenic molecules in the caudate nucleus and gray matter as mechanisms explaining BGC^7^. Neurological and psychological examinations as well as CT imaging remain the basic techniques for the diagnosis of Fahr’s syndrome^10^.

Treatment targets include symptoms and the identifiable cause, but there are no specific treatment limiting the progression of BGC^12^. Especially in HP, an early treatment can prevent calcification and neurophysiological disorders^12^. This case series corroborates the characteristics of Fahr’s syndrome as having indolent, silent, and oligosymptomatic outcomes in most cases. It underscores the importance of increasing physician awareness of best practices in the management of HP, including efforts to reduce large doses of oral calcium and active vitamin D while targeting a serum albumin‐corrected total calcium level near the lower limit of normal and maintaining 24‐hour urine calcium within the NR and a calcium phosphate product below 55 mg^2^/dL^2^ 3.
